# Decreased vaccination coverage and recurrence risk of measles due to COVID-19 pandemic

**DOI:** 10.17179/excli2021-4112

**Published:** 2021-08-30

**Authors:** Afrooz Mazidimoradi, Hamid Salehiniya

**Affiliations:** 1 Shiraz University of Medical Sciences, Shiraz, Iran; 2 Social Determinants of Health Research Center, Birjand University of Medical Sciences, Birjand, Iran

## ⁯⁯

***Dear Editor,***

World Health Organization (WHO) officially announced the COVID-19 pandemic in December in 2019 (Li et al., 2020[6]) which imposed additional burden on the public health system, therefore, epidemiological control of other infectious diseases has declined (Çavdaroğlu et al., 2021[2]; Phadke et al., 2021[8]).

On the other hand, outbreak of COVID-19 and constant advice to staying at home, fear of infection (Bode et al., 2021[1]), and suspension of childhood immunization programs, cause increased trend in other infectious diseases including measles (Rana et al., 2021[9]). Measles virus is a contagious disease that can be transmitted through direct contact through respiratory secretions, droplets or airborne transmission (Isa et al., 2012[5]).

During the pre-vaccination period, measles was endemic to the human population with every 2 to 3 years epidemics (Isa et al., 2012[5]). Most of the children got short-term illness; while some children with severe complications undergone permanent consequences such as blindness, deafness, mental disability or death (Vassantachart et al., 2020[11]). Nowadays, it is one of the most preventable diseases through vaccine injection and receiving one dose of measles vaccine that is given at 12 and 15 months, approximately has 93 % and 97 % efficacy respectively (Perrone and Meissner, 2020[7]).

Prior to the COVID-19 pandemic and by the end of 2019, 85 % of children aged 12-23 months worldwide had received the measles vaccine (World Bank, 2021[14]), but since the start of the COVID-19 pandemic, coverage has declined, and by 2020, nearly 120 million children in 37 countries had not received the measles vaccine (UNICEF, 2020[10]), many of whom live in Pakistan (Rana et al., 2021[9]).

In the United States from March 2017 to March 2020, the rate of 16-months-old children who received the MMR vaccine decreased from 72 % in March 2017 to 66.8 % in April to May 2020 (P <0.001) and then to 62.4 % (P = 0.02) from June to August 2020 (Bode et al., 2021[1]); while in order to achieve herd immunity to prevent the spread of measles, 93 to 95 % of the population should be vaccinated (Griffith et al., 2020[4]).

**Figure 1:**
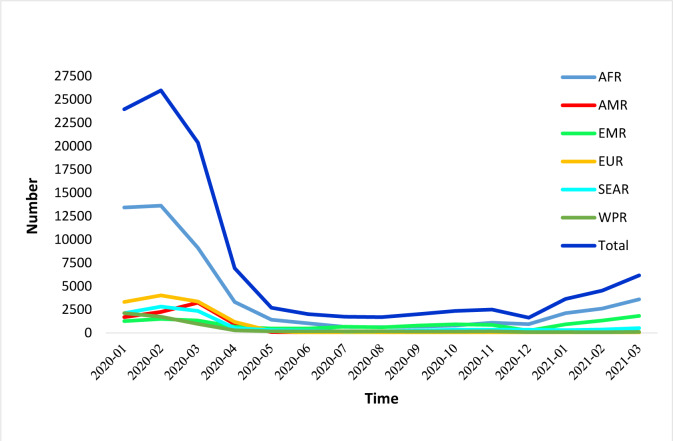
Number of reported measles cases by WHO regions

According to global statistics, the incidence rate of measles is increasing (Figure 1(Fig. 1)) (WHO, 2021[13]). Given the consequences of coronavirus, there is a concern that, despite the availability of a safe and effective vaccine, measles will again become a global challenge in young children and the plans to eradicate the disease will be disrupted. 

As demonstrated in WHO statistics (Figure 1(Fig. 1)) (WHO, 2017[12]), after the subsidence of the measles outbreaks in 2018 and 2019, again from the beginning of 2021, the world is witnessing an increasing trend in the incidence of measles, mainly in poor populations, which can be the result of reducing vaccination coverage due to COVID-19 pandemic.

Therefore, to prevent a new and deadly challenge among children, especially among poor countries that are major victims of COVID-19 (Durrheim et al., 2021[3]), the following measures are recommended:

- Retraining the primary care staff and general physicians about diagnose measles to improve diagnosis.
- Training of primary public health service providers forces in emergency situations such as Red Crescent on measles.
- Empowerment of health care systems in the field of monitoring and supervision of vaccination program, identification and isolation of patients and etc.
- Considering the high birth rate in poor countries, it is better to provide the cold chain equipment for vaccine storage for these countries by international organizations in order to increase vaccination coverage.
- Informing the public about the consequences of measles un-vaccination in children.
- Informing the public about the symptoms of measles in children through mass media and educational brochures in order to timely refer to medical centers to reduce the consequences of the disease.
- Strict implementation of health protocols to prevent COVID-19 infection (use of masks and observance of social distance and absence from crowded gatherings and centers), to prevent concurrent infectious with measles.
- Increased coverage of COVID-19 vaccination to prevent both infections at the same time
- Active follow-up of cases who did not receive measles vaccine as soon as possible.
- Separation of vaccine injection centers from other centers providing services related to COVID-19 disease.
- Implementation of scheduling programs in order to prevent the accumulation in vaccination centers.
- Strict implementation of health protocols in vaccination centers.
- Keep active the children's vaccination centers during the implementation of shutdown.
- Educating mothers about the importance of vaccination during pregnancy and postpartum by reproductive care providers in pregnancy care guidelines.

## Conflict of interest

The authors declare no conflict of interest.
